# Target Based Designing of Anthracenone Derivatives as Tubulin Polymerization Inhibiting Agents: 3D QSAR and Docking Approach

**DOI:** 10.1155/2014/658016

**Published:** 2014-04-17

**Authors:** Abdul Samad, Moawiah M. Naffaa, Mohammed Afroz Bakht, Manav Malhotra, Majid A. Ganaie

**Affiliations:** ^1^Department of Pharmaceutical Chemistry, College of Pharmacy, Salman bin Abdul Aziz University, P.O. Box 173, Al-kharj 11942, Saudi Arabia; ^2^Faculty of Pharmacy, University of Sydney, NSW 2006, Australia; ^3^Department of Pharmaceutical Chemistry, ISF College of Pharmacy, Ferozepur Road, Moga 142 001, India; ^4^Department of Pharmacology, College of Pharmacy, Salman bin Abdul Aziz University, P.O. Box 173, Al-kharj 11942, Saudi Arabia

## Abstract

Novel anthracenone derivatives were designed through *in silico* studies including 3D QSAR, pharmacophore mapping, and molecular docking approaches. Tubulin protein was explored for the residues imperative for activity by analyzing the binding pattern of colchicine and selected compounds of anthracenone derivatives in the active domain. The docking methodology applied in the study was first validated by comparative evaluation of the predicted and experimental inhibitory activity. Furthermore, the essential features responsible for the activity were established by carrying out pharmacophore mapping studies. 3D QSAR studies were carried out for a series of 1,5- and 1,8-disubstituted10-benzylidene-10H-anthracen-9-ones and 10-(2-oxo-2-phenylethylidene)-10H-anthracen-9-one derivatives for their antiproliferation activity. Based on the pattern recognition studies obtained from QSAR results, ten novel compounds were designed and docked in the active domain of tubulin protein. One of the novel designed compounds “N1” exhibited binding energy −9.69 kcal/mol and predicted Ki 78.32 nM which was found to be better than colchicine.

## 1. Introduction

Cancer is a leading cause of death worldwide and accounted for 7.6 million deaths (around 13% of all deaths) in 2008. The search for new anticancer drugs plays central role in the research programs of pharmaceutical companies and also for many governmental organizations [1]. Despite these efforts, the World Health Organization (WHO) estimates that the rate of incidence of such diseases will increase by 50% by the year 2020 [2]. It further projects that deaths from cancer worldwide may continue to rise over 11 million in 2030 [3]. For this reason, new and effective drugs are needed urgently.

In recent years, a large number of anticancer agents have been discovered to act at different levels [4] and have higher efficacy and lower toxicity than existing treatments. These databases can be exploited with the help of automated and multivariate data analysis methods [5, 6]. The later relates the molecular structures with their biological properties by establishing computational models able to assign activity values to new untested compounds [7, 8]. In the present study we have targeted microtubule polymerization for the inhibition of tumor cell growth. Microtubules are polymeric protein complexes constructed from a heterodimer of two highly homologous proteins known as *α*- and *β*-tubulin. The assembly of tubulin heterodimers into a macromolecular microtubule complex is a tightly regulated and dynamic process [9]. They are involved in a broad range of cellular processes, including the maintenance of cellular morphology and active transport of cellular components throughout the cytoplasm [10, 11]. In the mitotic phase of the cell cycle, microtubules are in dynamic equilibrium with tubulin dimers by assembling the tubulin into microtubules or, conversely, disassembling microtubules to tubulin [12]. Disruption of the dynamic equilibrium can induce cell cycle arrest and ultimately lead to apoptosis. Therefore, the compounds that could inhibit tubulin polymerization or interrupt microtubule depolymerization would be useful in the treatment of cancer.

In recent decades several compounds, mostly natural products, targeting tubulin have been discovered and developed; some of them are already in clinical use, such as epothilone, paclitaxel, and vindesine [13, 14]. Many of these agents exert their effects by inhibiting polymerization of tubulin to microtubules and almost all of them interact with the *α*- and *β*-tubulin dimer, rather than with microtubule-associated proteins (MAPs) or other proteins [15–18].

**Figure 1:**
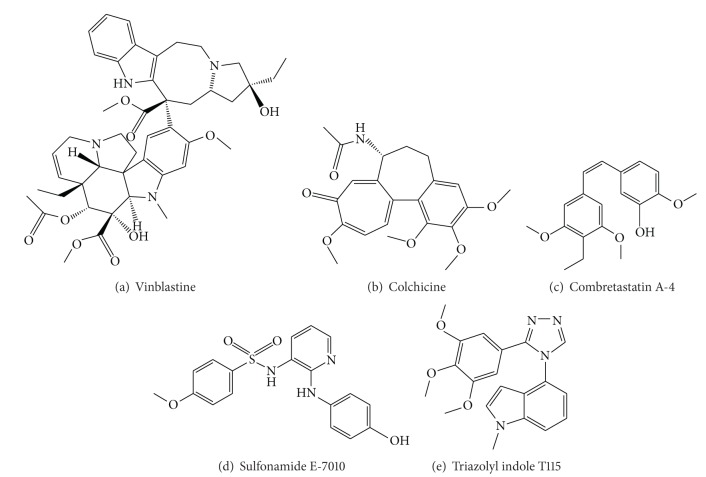
Reported tubulin interacting agents.

The Catharanthus bis-indole alkaloid vinblastine (Figure 1(a)) and the taxanes, such as paclitaxel and docetaxel, are important in the treatment of leukemias and lymphomas as well as many types of solid tumors. It was mainly the clinical success of these compounds that has stimulated intensive research aimed at additional microtubule-targeting drugs. The classic tubulin-binding agent colchicine (Figure 1(b)), which was purified from autumn crocus, has played an important role in elucidation of the properties and functions of tubulin and microtubules but has limited medicinal utility due to its high toxicity to mammalian cells. A structurally diverse collection of ligands, such as combretastatin A-4 [19] (Figure 1(c)) or the epothilones [20] as well as some synthetic molecules including sulfonamide E-7010 [21] (Figure 1(d)), triazolyl indole T115 [22] (Figure 1(e)), and so forth are known to exert cytotoxic activities through binding to tubulin.

Recently, tubulin polymerization inhibitors based on anthracenone scaffolds have been intensively investigated. A series of 10-(2-oxo-2-phenyl-ethylidene)-10H-anthracen-9-ones was described as potent inhibitors of tubulin polymerization [23]. These compounds were characterized by possessing an enone moiety between the anthracenone and the terminal aromatic ring. It is well known that the chemical and biological activities of anthraquinones, and related structural systems are greatly affected by the substitution pattern of the planar tricyclic ring system [24–29].

So in view of the above facts we hereby report the designing of some novel antitumor agents by targeting the inhibition of tubulin polymerization (ITP). This objective was achieved with the help of conventional and modern QSAR, pharmacophore mapping, and molecular docking studies from a series of 1,5- and 1,8-disubstituted10-benzylidene-10H-anthracen-9-ones and 10-(2-oxo-2-phenylethylidene)-10H-anthracen-9-one derivatives. In the present paper we account the pattern recognition studies through 3D QSAR and pharmacophore mapping studies. The insight for the binding mode of tubulin protein was established by docking of selected compounds of the series; for example, ITP values less than 1.8 *μ*M were docked into the binding pocket of tubulin using AutoDock 4.2 program. The rationale for novel designed compounds was established by docking of the newly designed compounds in the active domain and further through comparative analysis by considering colchicine as standard.

## 2. Methods and Material

### 2.1. Data Set

A series of compounds of 1,5- and 1,8-disubstituted10-benzylidene-10H-anthracen-9-ones and 10-(2-oxo-2-phenylethylidene)-10H-anthracen-9-ones were taken from the literature [23]. The compounds were subjected to the 3D QSAR studies for their antiproliferation activity. Selected representative compounds were further subjected to docking studies in the active domain of tubulin protein to validate the docking methodology.

There was high structural diversity and a sufficient range of the biological activity in the selected series of these derivatives (Table 1) which encourage us to select the data set for 3D QSAR and docking studies. The biological activity values (IC_50_ (nM)) were used as dependent variable.

**Figure 2:**
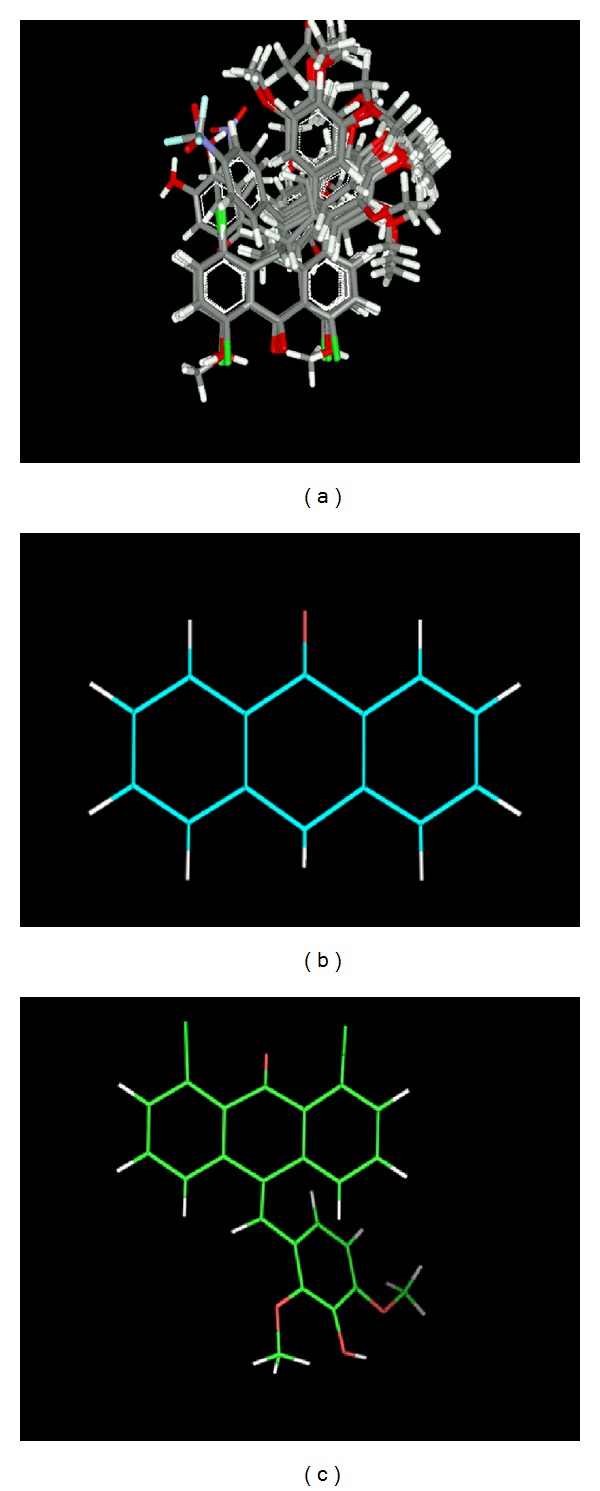
(a) Aligned molecules of the series. (b) Template Molecule for the alignment. (c) Reference molecule for the alignment.

All the compounds were built on 2D drawing workspace of molecular modeling software VLife MDS 3.5 (VLife Sciences Technologies Pvt. Ltd., Pune, India) and then the structure was exported to three-dimensional space for further analysis. The software was installed on Sony VAIO workstation having core 2 duo processor and windows 7 as operating system. All the molecules were batch optimized for minimization of energies using Monte Carlo conformational search with 10000 cycles [30] and Merck Molecular Force Field (MMFF) fields. All the 36 molecules of the series were aligned (Figure 2(a)) using template based alignment method by choosing a minimum common structure as “Template” (Figure 2(b)) and the most effective one as the “Reference Molecule” (Figure 2(c)). The goal was to obtain optimal alignment between the molecular structures necessary for alignment of compounds [31]. These aligned conformations were used to generate the predictive 3D QSAR models.

#### 2.1.1. 3D QSAR Methodology

For 3D-QSAR studies the aligned molecules of the series were exported to 3D-QSAR module worksheet. Activity of the molecules was fed in their respective columns. This was followed by the field computation of various electrostatic and steric descriptors available in the VLife descriptors list, which resulted in 2080 columns. The term descriptor has been utilized to indicate field values at the lattice points. The columns with fixed values were considered as Invariable columns and were deleted. The optimal training and test sets were generated using the sphere exclusion algorithm. This algorithm allows the construction of training sets covering descriptor space occupied by representative points. A training set of 28 molecules and a test set of 8 molecules were generated. Once the training and test sets were generated, the matrix with calculated descriptors was applied to one of the modest statistical treatment methods, that is, *k*NN-MFA (*k*-Nearest Neighbor Molecular Field Analysis). In this methodology the antiproliferation activity of the compounds was taken as dependent variable and the rest of the columns were considered as independent variable.

Like many 3D QSAR methods *k*-Nearest Neighbor molecular field analysis (*k*NN-MFA) requires suitable alignment of given set of molecules. This was followed by generation of a common rectangular grid around the molecules. The steric and electrostatic interaction energies were computed at the lattice points of the grid using a methyl probe of charge (+1). These interaction energy values were considered for relationship generation and utilized as descriptors to decide nearness between molecules.

### 2.2. Feature Selection and Model Development

Feature selection is a key step in QSAR analysis. An integral aspect of any model-building exercise is the selection of an appropriate set of features with low complexity and good predictive accuracy. This process forms the basis of a technique known as feature selection or variable selection [32]. Among several search algorithms, stepwise (SW) forward-backward variable selection method, genetic algorithms (GA), and simulated annealing (SA) based feature selection procedures are most popular for building QSAR models and can explain the conformational preference more effectively. In search of the best models with improved *q* ^2^ and pred_*r* ^2^ values, each of the variable selection methods was attempted with the optimal changes in statistical parameters until we got an acceptable 3D QSAR model. The three satisfactory 3D QSAR models (A, B, and C) generated by changing the statistical parameters have been reported here.

“Stepwise Forward-Backward” process was chosen as variable selection method for “Model A” along with cross correlation limit 0.5, variances cut-off zero, and “mean centering” as scaling method for the development of this model. Further, for *k*-Nearest Neighbor parameters, maximum and minimum number of neighbors was set as 9 and 2, respectively, along with “*k*NN classification” as the prediction scheme.

For Model B “Simulated annealing” process was chosen as variable selection method along with cross correlation limit as 0.5, terms in model as 4, maximum temperature as 100, minimum temperature as 0.01, iteration at given temperature as 5, variance cut-off zero, and “mean centering” as scaling method for the development of this model.

“Stepwise Forward-Backward” method was chosen as variable selection method for “Model C” along with cross correlation limit 0.5, variance cut-off two, and “None” as scaling method for the development of this model. Further, for *k*-Nearest Neighbor parameters, maximum and minimum numbers of neighbor were set as 9 and 2, respectively, along with “Distance based weighted average” as the prediction method.

#### 2.2.1. Pharmacophore Mapping Studies

Generating a pharmacophore is usually the first step for understanding the interaction between a receptor and a ligand. Over the years, pharmacophores have been successfully used in lead generation, scaffold hopping, mining small molecule databases, and so forth [33, 34]. The pharmacophore tools can be used to build pharmacophore models from a ligand, receptor, or receptor-ligand complex. The tools can also be used to analyze pharmacophore-ligand interactions, analyze pharmacophore similarity, build and mine databases, screen ligand libraries, and customize pharmacophore features.

Pharmacophore modeling experiment was carried out to develop a hypothetical pharmacophore model for the antitumor activity aiming to study the fitting of the series of molecules under study. Pharmacophore mapping was carried out through the Molsign package of VLife MDS 3.5 software. The pharmacophore model was developed by choosing training set of most active anthracenone derivatives including compounds 16f, 16g, 16h, 31a, 31b, and 31c.

## 3. Docking Studies

We have carried out molecular docking studies for selected compounds of 1,5- and 1,8-disubstituted10-benzylidene-10H-anthracen-9-ones and 10-(2-oxo-2-phenylethylidene)-10H-anthracen-9-ones with the tubulin protein. Crystal structures of tubulin protein in complex with colchicine (PDB ID: 1SA0) [35] with resolution 3.5 Å was downloaded from RCSB Protein Data Bank to serve as the docking template. The crystallographic water and ligand molecules were removed from the tubulin complex. Docking studies were carried out on AutoDock 4.2 [36], running on Linux Ubuntu 10.0, installed on Pentium i3 workstation. ChemDraw ultra 8.0 software (Chemical Structure Drawing Standard; Cambridge Soft Corporation, USA (2003)) was used for construction of compounds which were converted to 3D structures using Chem3D ultra 8.0 software and the constructed 3D structures were energetically minimized by using MOPAC (semiempirical quantum mechanics) with AM1 mozyme geometry, 100 iterations, and minimum RMS gradient of 0.10. The program AutoDock Tools (ADT) released as an extension suite to the Python Molecular Viewer was used to prepare the protein and the ligand to convert the molecules into AutoDock type, which is a prerequisite for the docking [37]. Pymol software [38] was used for visualization purposes of docked confirmations.

All the receptor and ligand files were converted to “pdbqt” format, which was pdb plus “q” charges and “t” AutoDock type. For the macromolecule, polar hydrogens were added, and then Kollman United Atom charges and atomic solvation parameters were assigned. For the ligand, hydrogens were added before computing Gasteiger charges, and then the nonpolar hydrogens were merged. For each ligand, corresponding ATOM/HETATM and CONECT records were extracted from protein complex in pdb file. After assigning bond orders, missing hydrogen atoms were added. Then in the AutoDock tools package, the partial atomic charges were calculated using Gasteiger-Marsili method [39] and after merging nonpolar hydrogens, rotatable bonds were assigned. All amide bonds considered nonrotatable. For receptor, the ligand, as well as any additional chains, and all the heteroatoms including water molecules were removed. By the use of AutoDock Tools all missing hydrogens were added. Input molecules files for AutoDock experiments must conform to the set of atom types supported by it. Therefore, pdbqt format was used to write ligands, recognized by AutoDock. The grid maps were calculated using AutoGrid [40]. In all dockings, a grid map with 66 × 66 × 66 points and a grid spacing of 0.503 Å were used, and the maps were centered on the ligand binding site.

Of the three different search algorithms offered by AutoDock 4.2, the Lamarckian genetic algorithm (LGA) based on the optimization algorithm [41] was used, which utilizes (discredited) Lamarckian notation in which adaptations of an individual to its environment can be inherited by its offspring. For all dockings, the default values for all the parameters were used. AutoDock 4.2 was used to generate both grid and docking parameter files (.gpf and  .dpf files). The docking results from each calculation were clustered on the basis of root-mean square deviation (RMSD) between the Cartesian coordinates of the ligand atoms and were ranked according to the binding free energy. The structure with relative lower binding free energy and the most cluster member was chosen for the optimum docking conformation. For each docking experiment, the lowest energy docked structure was selected from 100 runs. In order to evaluate accuracy of docking, binding energy, root mean square positional deviation (RMSD), and numbers in cluster were used. Ki values (nM) were recorded for the lowest binding energy mode. Lower binding free energy and lower Ki values along with more numbers of clusters were considered as the criteria of evaluation.

Our most active, selected representative compounds (16c, 16f, 16h, 20c, 20i, and 31a–e) were modeled by positioning them in the colchicine (PDB ID: 1SA0) binding site in accordance with the published crystal structure of colchicine bound in the domain of chains A and B. The entire complex was then subjected to alternate cycles of minimization and dynamics. The intent was to get a satisfactory structure for the complex that was consistent with the published crystal structure. From the comparative docking study of our compounds with standard binding compound (colchicine) we could observe how our compounds might bind to the polymer inhibition site, based on the knowledge of the structure of similar active sites. We redocked colchicine into the active site of the protein between chains A and B (Figure 5) and then we docked with our compounds in order to compare the binding mode of both ligand and the test compound.

## 4. Results and Discussion

The importance and utility of 3D QSAR studies have been established by applying it to known sets of molecules as described above. In the present paper we further support our study by pharmacophore mapping and docking of the most active representative compounds in the active domain of tubulin protein. In this study our main focus is to establish pattern recognition of anthracenone derivatives for their antiproliferation activity and to further design the novel compounds with better binding affinity.

### 4.1. 3D QSAR Analysis

Several 3D QSAR models were generated by *k*NN-MFA in conjunction with Simulated Annealing (SA), Genetic Algorithms and Stepwise (SW) Forward Backward selection methods, and the corresponding few best models generated were reported.

The training and test set for 3D QSAR studies were distributed by random selection method. To ensure a fair comparison, the same training and test sets were used for each model's development. Some statistically significant models as shown in Table 2 have been considered to be discussed here. The term selection criterion for the best model was set by choosing maximum *q* ^2^ value, pred_*r* ^2^ value, and the optimum number of descriptors. The steric (S), electrostatic (E), and hydrophobic (H) descriptors specify the regions, where variation in the structural features of different compounds in the training set leads to increase or decrease in activities. The numbers accompanied by the descriptors represent its position in the 3D MFA grid.

In model A we have found the *q* ^2^ value =** 0.8330 **and pred_*r* ^2^ =** 0.7456 **along with three steric descriptors that has indicated the internal predictive power of the model 83.3% and external prediction 74.5%. The *q* ^2^_se = 1.8935 (*q* ^2^ standard error) was also found to be in a veryacceptable range which further indicated its significance in the prediction and development of new anthracenone derivatives as novel anticancer compounds.

**Figure 3:**
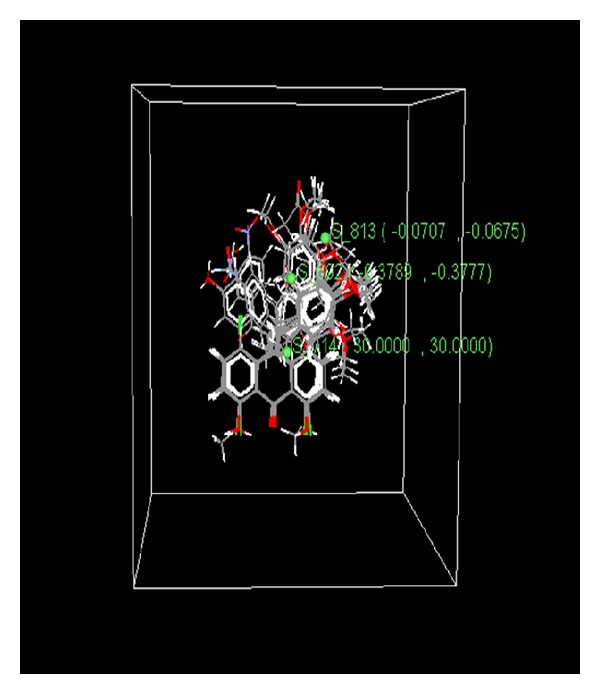
3D QSAR model (*k*NN-MFA plot) indicating relative position of descriptors by green solid sphere.

Plot of the *k*NN-MFA (Figure 3) shows the relative position and ranges of the corresponding important electrostatic and steric fields. The negative range of descriptor S_692 (−0.3789 to −0.3777) indicates that in this region negative steric potential was favorable for increase in the activity and hence less bulky substituent would be preferred to enhance the activity. In the same way another descriptor S-813 (−0.0707 to −0.0675) indicated that less bulky substituent would be suitable to attain the optimum activity as the molecules had already bulky substitution in this region. On the other hand, descriptor S_714 having positive steric potential (30) was found canvassing for more bulky group substitutions in that region to enhance the activity.

Model B was found to have the *q* ^2^ value =** 0.8008 **and pred_*r* ^2^ =** 0.6923** along with one steric and three electronic descriptors that indicated the internal predictive power of the model 80% and for external prediction 69.23%. The *q* ^2^_se = 2.0682 (*q* ^2^ standard error) was also found to be in acceptable range which further supports the model to be statistically significant. Further, for *k*-Nearest Neighbor parameters, maximum and minimum numbers of neighbor were set as 5 and 2, respectively, along with “*k*NN classification” as the prediction method. The steric descriptor in the model S_957 having negative potential (−0.3844 to −0.3073) indicating that less bulky substituent will be preferred in that region for the favorable activity. The rest of the two electronic descriptors have electrostatic potential in the positive range (E_1422: 0.1359 to 0.2768 and E_213: 0.0427 to 0.7289) and the other one has range (E_448: −2.8750 to 0.5249). So this model favored the less electronegative as well as less bulky substituent at the above mentioned points.

Model C was found to have the *q* ^2^ value = 0.7722 and pred_*r* ^2^ = 0.8243 along with two electronic and two steric descriptors that indicates the internal predictive power of the model 72.22% and for external validation 82.43%. The *q* ^2^_se = 2.2119 (*q* ^2^ standard error) was also found to be in the acceptable range which proves this model to be significant for the prediction purposes of novel anticancer compounds. The steric descriptor S_1121 has negative electrostatic potential range from −0.7512 to −0.6729 indicating the presence of less bulky substituent favorable for the activity. Another descriptor S_953 (2.3057 to 15.4769) indicated the need of more bulky group substituents for the better activity. The electronic descriptor E_1113 having potential (−3.7570 to −3.6936) indicated that less electronegative substituent would be more favorable for the activity. Another electronic descriptor E_583 having electrostatic potential 10.0 urged for the less electronegative substituent for the optimum activity. From Table 3 it is evident that the predicted activities of all the compounds in the test set are in good agreement with their corresponding experimental activities and optimal fit has been obtained.

Among the 3 selected models, the best one was chosen by the comparative study of Table 3. Model A has the highest *q* ^2^ value (0.8330), least *q* ^2^_se (1.8935), and appreciable pred_*r* ^2^ (0.7456); but model C has *q* ^2^ value (0.7722), *q* ^2^_se (2.2119), and the best pred_*r* ^2^ (0.8243) that makes model A to be considered the best with respect to the above parameters. So model A can be chosen as well thought out for SAR studies, pattern recognition, and prediction purposes.

#### 4.1.1. Pharmacophore Mapping

**Figure 4:**
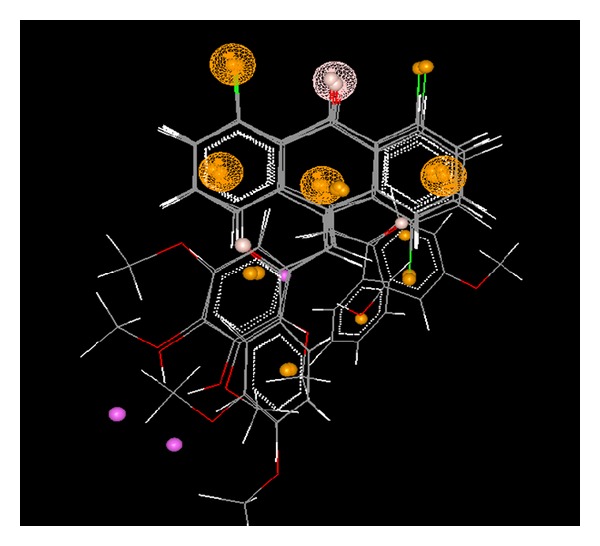
Pharmacophore model representing the essential feature by large tessellated sphere. The coloring scheme for the various large tessellated spheres is as follows: hydrogen bond donor: magenta color; hydrogen bond acceptor: buff color; hydrophobic: orange color; aliphatic: orange color.

The generated hypothetical pharmacophore (Figure 4) showed five overlapping points with similar chemical properties in the training set. The mapping was based on these points; two aliphatic carbons, two aromatic carbons, and one H acceptor. The larger tessellated spheres were indicative of the common pharmacophoric features identified in the molecules, and the smaller solid features were for the individual molecules. The pharmacophoric features shown by the tessellated spheres were indicative of the necessary groups needed for the optimum activity. Furthermore, it added the support for structural activity relationship by giving the evidence that both aromatic rings were necessary for activity along with one chorine group and carbonyl group joining both phenyl rings. This fact was also well supported by the reported activity that compounds with chlorine substitution had better activity when replaced with hydroxyl group. Moreover the model depicted that the carbon atom joining the phenyl rings at the other end was also a necessary part of pharmacophore. The coloring scheme for the various large tessellated spheres was as follows: hydrogen bond donor: magenta color; hydrogen bond acceptor: buff color; hydrophobic: orange color; aliphatic: orange color; negative ionizable: green color; positive ionizable: violet color. From the model it is evident that the fitting pattern of the antheracenone derivatives was supported by the pharmacophore studies of the series.

#### 4.1.2. Molecular Docking Studies

**Figure 5:**
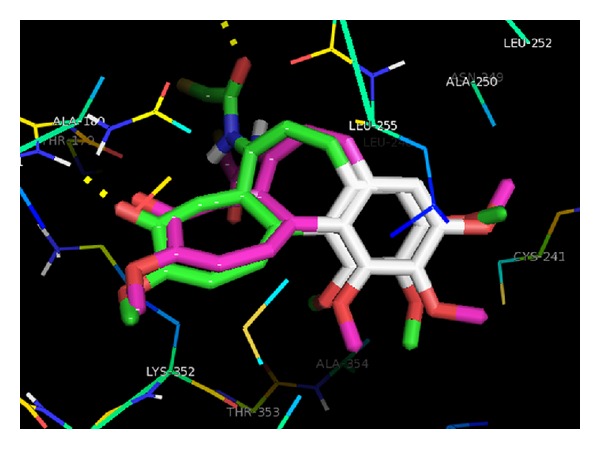
Redocked structure of colchicine, overlapped with the cocrystallized one.

*Molecular Docking Analysis for Representative Anthracenone Derivatives.* The docking studies provided us the insight into structural relation of anthracenone derivatives with the inhibition of tubulin polymerization (ITP) through the binding domain of tubulin-colchicine complex. Docking method was validated by redocking the colchicine with the tubulin protein and the interactions obtained were considered as the standard to compare with the docking of selected compounds. Redocked structure of colchicine as shown in Figure 5 revealed that hydrogen bonding interaction with the residues Val-181 and Asn-101 was the key feature of the successful molecules for ITP. The binding pattern of colchicine was considered as the requisite for a molecule to be active.

**Figure 6:**
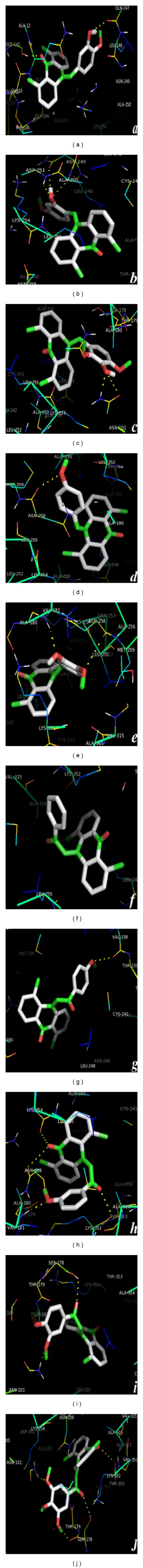
Visualization of docking mode of selected anthracenone derivatives. Yellow dashed lines are representing the hydrogen bonding as well as polar interactions.

Though the anthracenone derivatives have different structural scaffold but even the selected docked compounds were found to have H-bond interactions either with Val-181 or Asn-101. The docked compounds showed comparable binding affinity, which was further in compliance with the mode of action and binding mode for colchicine. The binding affinities were in the range of −8.98 to −7.91 kcal/mol as shown in Table 4. Docked conformations of compounds 16h, 31d, and 31e showed interaction with the residue Asn-101 (Figures 6(c), 6(i), and 6(j), resp.). On analyzing the structure of these 3 test compounds it gave us an idea that the methoxy substitution of phenyl ring at* meta* position was responsible for the H-bond interaction. Compounds 16f, 20c, and 31b have methoxy substitution at para position but they were showing H-bond interaction with different residues, further conforming to the significance of methoxy group at* meta* position.

Though 16f was bound to Asp-251 (Figure 6(b)) rather than Val-181 or Asn-101 but even it exhibited good activity; so the reason could be the polar interactions of molecule with Ala-250 and Asn-249. Compounds 20i and 31c both exhibited H-bond interaction with the residue Val-181 (Figures 6(e) and 6(h)) with high potency, thereby establishing the importance of Val-181 residue for the activity. The close analysis concluded that methoxy substitution at* meta* position in the phenyl ring in compound 31c resulted in H-bond interaction with residue Val-181. Compound 31c also has the significant polar interactions with three residues, namely, Lys-254, Asn-258, and Lys-352, further giving the explanation for its highest binding energy (−8.98 kcal/mol) and lowest inhibition constant (259.6 nM), making it highly potent in the series. Although compound 20i had methoxy substitution at para position but interestingly it showed H-bond interaction with residue Val-181 conferring it high potency, the credit for this bonding went to the presence of adjacent hydroxyl group at* ortho* position. Compound 16f also had methoxy substitution at* para* position and hydroxyl group at* ortho* position but it did not show H-bonding with Asn-101; the reason could be the absence of electronegative carbonyl group and the distance of hydroxyl group from methoxy group. Compound 31a did not show any H-bond interaction (Figure 6(f)) which indicated the reason that there was no methoxy group, instead it had polar and van der Waals interactions which led it to have good binding affinity (−8.62 kcal/mol) as well as predicted inhibition constant (482.67 nM), thereby resulting into good activity. One important reason for the binding of the molecules with Val-181 or Asn-101 was observed that when they had di or tri methoxy substitution (16h, 31d, and 31e) they exhibited binding with Asn-101 (Figures 6(c), 6(i), and 6(j), resp.) while only one methoxy substituted compound (20c) exhibited binding with Asn-258 (Figure 6(d)). Compound 31b stands second after 31c with respect to the free binding energy as well as for predicted inhibition. It showed no hydrogen bonding but the polar interaction with Val-238 (Figure 6(g)). Furthermore, compounds 31a and 31b were showing appreciable binding affinity with polar interactions only but without any H-bond interaction. By this comparative analysis of binding mode of colchicine and the series of compounds under the study we have attempted to identify the important residues responsible for ITP.

*Molecular Docking Analysis for the Novel Designed Compounds.* Based on the understanding of 3D QSAR results, binding pattern of colchicine and selected docked compounds, we have designed some novel compounds which were further docked with the tubulin protein.

**Figure 7:**
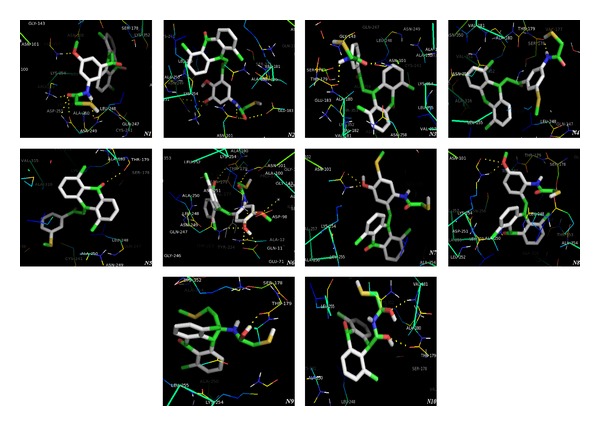
Visualization of docking mode of novel anthracenone compounds. Yellow dashed lines are representing the hydrogen bonding as well as polar interactions.

The best binding affinity (−9.69 kcal/mol) and predicted polymer inhibition constant (78.32 nM) were shown by compound N1. The carbonyl group present in the side chain of molecule had shown H-bond interaction with Leu-248 and polar interaction with Asn-249 residue belonging to chain B. Moreover N1 also exhibited H-bonding with Asn-101, Asp-251, and Lys-254 residues (Figure 7-N1). Compound N2 exhibited moderate binding affinity −8.26 and predicted polymer inhibition constant 739.94 nM. It made H-bond interaction with Asn-101 and LYS-254, imparting good binding affinity for the active domain. It also exhibited polar interaction with Asn-249, Glu-183, and Asn-101 residues (Figure 7-N2). Compounds N3 and N4 have shown binding affinity −8.91 kcal/mol and −8.81 kcal/mol with good predicted activity 296.44 nM and 348.65 nM, respectively. The H-bond interaction was observed with Asn-101 and Thr-179 residues (Figure 7-N3). Compound N5 exhibited an important polar interaction between carbonyl group of anthracenone moiety and carbonyl group of Thr-179 residue (Figure 7-N5); in contrast to other compounds it has not shown any H-bond interaction. Compound N6 has shown moderate binding affinity −8.36 kcal/mol and predicted polymer inhibition constant 744.37 nM. N6 also had H-bond interaction with Asn-101, Asn-249, and Lys-254, imparting good binding affinity for the active domain. It also exhibited polar interaction with Glu-71 and Thr-179 residues (Figure 7-N6). Compounds N7 and N8 have shown moderate binding affinities −8.23 and −8.48 kcal/mol, respectively, and both also exhibited H-bond interaction with Asn-101. Further from compound 8, methoxy substitution has been proved more beneficial for the activity (Figures 7-N7 and 7-N8). Compounds N9 and N10 were also found to bind with poor binding affinities showing poor binding free energy which further supported our pharmacophore mapping model conforming that phenyl ring attached to anthracenone moiety was essential for the activity (Figure 7, N9 and N10).

## 5. Conclusion

To establish a rational for SAR study, 3D QSAR studies were carried out for the anthracenone derivatives taken into study. Pharmacophore mapping studies were carried out for the development of essential features required for the activity. Further to understand the mechanism of binding of tubulin heterodimer with the ligands, molecular docking studies were carried out with the selected representative anthracenone derivatives. The present work has led to the designing of novel tubulin inhibitor molecules and some of which have shown promising activities. The modern QSAR and pharmacophoric studies have established the following points: (1) both hydrophobic rings present in anthracenone moiety are essential for the activity; (2) carbonyl group attached to the anthracene moiety has been found as essential part of the pharmacophore model; (3) chlorine atom at position one is highly required for the activity; (4) less bulky substituents are required at the carbon just linking with the anthracene and optimum bulky groups at the phenyl moiety attached; and (5) electronic descriptor E_583 having electrostatic potential 10.0 urges for the less electronegative substituent for the optimum activity.

Docking studies with the representative anthracenone derivatives explored that H-bonding of the ligand with Val-181 and Asn-101 residues plays a very important role in inhibition of tubulin polymerization. Docked conformations of compounds 16h, 31d, and 31e explored that di- or tri-methoxy substitution is favorable for the inhibition. In addition methoxy substitution at* meta* position was favored more for the activity. Residue Val-238 was explored in this study as an important one because the compound (31b), having interaction with Val-238, has shown good binding affinity. On the basis of these insights obtained from the molecular modeling studies, ten novel molecules were designed and docked with the tubulin protein. One of our newly designed molecules N1 has been found very promising as it exhibited excellent free binding energy −9.69 (kcal/mol) and predicted inhibition constant (Ki) 78.32 nM even better than colchicine. Compounds N3, N4, N6, and N8 have also exhibited comparable binding affinity as well as predicted activity (Table 5). From our studies we have found some residues such as Thr-179, Ser-178, Lys-254, and Asn-249 important for the activity as the molecules interacting with them have shown good activity as well as binding affinity. So these residues need to be explored more in future to find the better interacting agents in the active domain of tubulin protein. The work on anthracenone derivatives is ongoing in our lab for further understanding the structure activity relationship and binding mode with tubulin protein in order to obtain more ligands with better antitumor activity and less toxicity. In future these promising novel compounds will be subjected for wet lab synthesis after extensive* in silico *investigation.

## Figures and Tables

**Table 1:** Structure of the molecules taken into study.

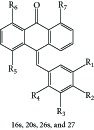 * * 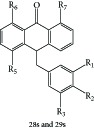 * * 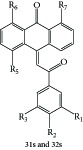										
Serial number	CPD	R_1_	R_2_	R_3_	R_4_	R_5_	R_6_	R_7_	K562	ITP
									(IC_50_ μM)	(IC_50_ μM)

1	16a	H	OCH_3_	H	H	H	Cl	Cl	0.61	0.87
2	16b	OCH_3_	OCH_3_	OCH_3_	H	H	Cl	Cl	10	>10
3	16c	OH	OCH_3_	H	H	H	Cl	Cl	0.25	0.36
4	16d	OCH_3_	OH	H	H	H	Cl	Cl	5	6.8
5	16e	H	H	OCH_3_	OH	H	Cl	Cl	5	>2 μM
6	16f	H	OCH_3_	H	OH	H	Cl	Cl	0.5	1.6
7	16g	OCH_3_	OH	OCH_3_	H	H	Cl	Cl	2.0	1.66
8	16h	H	OCH_3_	OH	OCH_3_	H	Cl	Cl	0.10	0.36
9	20c	H	OCH_3_	H	H	Cl	H	Cl	1.40	0.69
10	20e	OCH_3_	OCH_3_	OCH_3_	H	Cl	H	Cl	18	ND
11	20i	OH	OCH_3_	H	H	Cl	H	Cl	0.21	0.42
12	26a	H	H	H	H	H	OH	OH	2.3	>10
13	26b	H	OCH_3_	H	H	H	OH	OH	2.0	4.0
14	26c	OCH_3_	OCH_3_	H	H	H	OH	OH	2.0	>10
15	26d	OCH_3_	OCH_3_	OCH_3_	H	H	OH	OH	2.6	>10
16	26e	H	OH	H	H	H	OH	OH	2.0	ND
17	26f	OH	OH	H	H	H	OH	OH	2.6	ND
18	26h	OH	OCH_3_	H	H	H	OH	OH	1.50	1.93
19	26i	H	CF_3_	H	H	H	OH	OH	1.7	ND
20	26j	H	NO2	H	H	H	OH	OH	1.7	>10
21	27	H	OCH_3_	H	H	H	OCH_3_	OCH_3_	1.42	>2
22	28c	OH	OCH3	H	—	H	Cl	Cl	1.60	1.20
23	29b	H	OCH_3_	H	—	Cl	H	Cl	13	ND
24	29c	OCH_3_	H	H	—	Cl	H	Cl	15	ND
25	29f	OH	OCH_3_	H	—	Cl	H	Cl	5.10	ND
26	31a	H	H	H	—	Cl	H	Cl	0.29	1.70
27	31b	H	OCH_3_	H	—	Cl	H	Cl	0.22	0.53
28	31c	OCH_3_	H	H	—	Cl	H	Cl	0.25	0.69
29	31d	OCH_3_	OCH_3_	H	—	Cl	H	Cl	0.24	0.69
30	31e	OCH_3_	OCH_3_	OCH_3_	—	Cl	H	Cl	0.23	0.36
31	31f	H	Cl	H	—	Cl	H	Cl	1.60	ND
32	31g	H	Br	H	—	Cl	H	Cl	1.70	0.51
33	31h	H	NO_2_	H	—	Cl	H	Cl	1.90	ND
34	31i	H	OCOCH_3_	H	—	Cl	H	Cl	3.4	0.41
35	32a	H	OCH_3_	H	—	H	Cl	Cl	0.93	0.73
36	32b	OCH_3_	OCH_3_	OCH_3_	—	H	Cl	Cl	0.72	1.62

**Table 2:** 3D QSAR model summary for statistically significant models.

Statistical parameter	Model A	Model B	Model C
Training set size	28	28	28
Test set size	8	8	8
k-Nearest Neighbor	2	5	2
*n*	28	28	28
Degree of freedom	24	23	23
*q* ^2^	**0.8330**	**0.8008**	**0.7722**
*q* ^2^_se	1.8935	2.0682	2.2119
Pred *r* ^2^	**0.7456**	**0.6923**	**0.8243**
Pred_*r* ^2^ se	1.2660	1.3923	1.0521
Descriptor	S_692	E_448	S_1121
Range	−0.3789	−2.8750	−0.7512
	−0.3777	0.5249	−0.6729
	S_813	S_957	E_583
	−0.0707	−0.3844	10.0000
	−0.0675	−0.3073	10.0000
	S_714	E_1422	S_953
	30.0000	0.1359	2.3057
	30.0000	0.2768	15.4769
		E_213	E_1113
		0.0427	−3.7570
		0.7289	−3.6936

**Table 3:** Experimental and predicted activity by statistically significant 3D QSAR models.

Serial number	CPD	Experimental activity	3D QSAR		
			Model A	Model B	Model C
1	16a	0.61	0.954863	1.883334	0.557213
2	16b	10	11.499623	7.132195	9.999986
3	16c	0.25	0.269992	1.090947	0.673721
4	16d	5	3.800172	1.971551	4.94837
5	16e	5	3.150917	1.865987	5.00000
6	16f	0.5	0.35503	1.849256	0.552787
7	16g	2	1.050406	1.941135	1.385509
8	16h	0.1	1.049594	1.84818	0.160045
9	20c	1.4	0.955137	6.661933	1.623963
10	20e	18	15.152435	10.58147	17.299996
11	20i	0.21	1.40481	5.586993	1.042793
12	26a	2.3	2.150009	2.304161	2.173967
13	26b	2	1.750454	3.292628	1.167205
14	26c	2	1.015052	3.130678	2.307389
15	26d	2.6	3.799828	3.387593	2.553173
16	26e	2	2.149991	2.301159	2.126033
17	26f	2.6	2.300034	2.288864	2.420742
18	26h	1.5	1.460009	3.309645	0.987624
19	26i	1.7	1.700000	1.887163	1.700000
20	26j	1.7	1.700000	1.81553	1.700000
21	27	1.42	1.459991	1.87583	1.05444
22	28c	1.6	0.945001	1.686606	0.763163
23	29b	13	11.500377	10.46197	13.091177
24	29c	15	16.14408	7.330496	14.908823
25	29f	5.1	3.851212	4.275086	4.54596
26	31a	0.29	0.944996	5.508271	0.863511
27	31b	0.22	0.969919	1.652002	1.339498
28	31c	0.25	0.961289	0.397121	0.16449
29	31d	0.24	0.943851	0.394247	0.188792
30	31e	0.23	0.602966	0.95067	0.169955
31	31f	1.6	0.945004	5.805245	1.026488
32	31g	1.7	0.945002	5.826295	0.986579
33	31h	1.9	1.750006	5.900798	1.771209
34	31i	3.4	0.772665	1.069587	1.245819
35	32a	0.93	1.700000	1.900265	1.339195
36	32b	0.72	0.964958	1.363822	1.332919

**Table 4:** Docking summary for the selected eligible anthracenone derivatives.

CPD	IPT IC_50_ (*μ*M)	Binding energy (kcal/mol)	Ki predicted activity	Torsional energy (kcal/mol)	H-bonding “residue”
16c	0.36	−8.26	882.37 nM	0.89	Ser-140
					Gln-247
16f	1.60	−8.22	945.09 nM	0.89	Asp-251
16h	0.36	−8.07	1.22 uM	1.19	Asn-101
20c	0.69	−8.73	398.87 nM	0.6	Asn-258
20i	0.42	−8.80	351.79 nM	0.89	Val-181
31a	1.70	−8.62	482.67 nM	0.6	—
					(only polar interactions)
31b	0.53	−8.91	293.25 nM	0.89	Val-238
31c	0.69	−8.98	259.6 nM	0.89	Val-181 NH
31d	0.69	−8.16	1.04 uM	1.19	Asn-101
					Ser-178
31e	0.36	−7.91	1.6 uM	1.49	Asn-101
					Ser-178
Colchicine	1.40	−9.47	114.73 nM	1.79	Val-181
					Asn-101

**Table 5:** Docking summary for the designed compounds.

Compound	Structure	Binding energy (kcal/mol)	Ki predicted activity	Torsional energy (kcal/mol)	H-bonding “residue”
N1	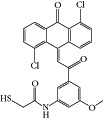	−9.69	78.32 nM	1.79	Asn-101 Asp-251 Asn-249 Leu-248

N2	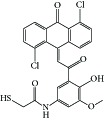	−8.36	739.94 nM	2.09	Asn-101 Lys-254

N3	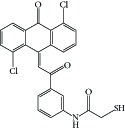	−8.91	296.44 nM	1.49	Asn-101 Thr-197

N4	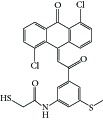	−8.81	348.65 nM	1.79	Ser-178

N5	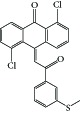	−8.26	881.59 nM	0.89	— (only polar interactions)

N6	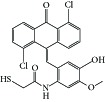	−8.36	744.37 nM	1.79	Asn-101 Lys-254 Asn-249

N7	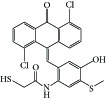	−8.23	928.62 nM	1.79	Asn-101

N8	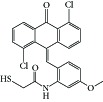	−8.48	611.75 nM	1.49	Asn-101

N9	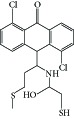	−7.03	7.0 μM	2.68	Thr-179

N10	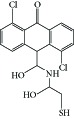	−6.81	10.24 μM	2.09	Val-181
